# miRNA on the Battlefield of Cancer: Significance in Cancer Stem Cells, WNT Pathway, and Treatment

**DOI:** 10.3390/cancers16050957

**Published:** 2024-02-27

**Authors:** Lekha Bhagtaney, Arun Dharmarajan, Sudha Warrier

**Affiliations:** 1 Division of Cancer Stem Cells and Cardiovascular Regeneration, Manipal Institute of Regenerative Medicine, Manipal Academy of Higher Education (MAHE), Bangalore 560065, India; lekhabhagtaney@gmail.com; 2 Faculty of Clinical Research, Sri Ramachandra Institute of Higher Education and Research, Chennai 600116, India; 3 School of Human Sciences, Faculty of Life and Physical Sciences, The University of Western Australia, Perth, WA 6009, Australia; 4 Curtin Medical School, Faculty of Health Sciences, Curtin University, Perth, WA 6102, Australia; 5 Department of Biotechnology, Faculty of Biomedical Sciences and Technology, Sri Ramachandra Institute of Higher Education and Research, Chennai 600116, India; 6 Cuor Stem Cellutions Pvt Ltd., Manipal Institute of Regenerative Medicine, Manipal Academy of Higher Education (MAHE), Bangalore 560065, India

**Keywords:** cancer stem cells, WNT signaling pathway, microRNA, miRNA therapeutics

## Abstract

**Simple Summary:**

The development of cancer proceeds when the intricate dynamics of the cell are destabilized. It is a multifaceted process, and the involvement of one particularly notorious cell type, cancer stem cells, has been increasingly observed. The WNT signaling pathway is a prominently deregulated pathway in cancer stem cells and has been suggested to maintain properties of self-renewal, tumor initiation, metastasis, and chemoresistance in these cells. MicroRNAs (miRNAs) are post-transcriptional gene regulators that can directly influence gene expression in cancer. As miRNAs hold the potential to be either oncogenic or tumor suppressors, they are found to be highly dysregulated in numerous cancers. Understanding the function of miRNAs in the context of cancer stem cells and the WNT signaling pathway may provide new insights into the treatment of cancer, which is a leading cause of death worldwide.

**Abstract:**

Carcinogenesis is a complex process characterized by intricate changes in organ histology, biochemistry, epigenetics, and genetics. Within this intricate landscape, cancer stem cells (CSCs) have emerged as distinct cell types possessing unique attributes that significantly contribute to the pathogenesis of cancer. The WNT signaling pathway plays a critical role in maintaining somatic stem cell pluripotency. However, in cancer, overexpression of WNT mediators enhances the activity of β-catenin, resulting in phenomena such as recurrence and unfavorable survival outcomes. Notably, CSCs exhibit heightened WNT signaling compared to bulk cancer cells, providing intriguing insights into their functional characteristics. MicroRNAs (miRNAs), as post-transcriptional gene expression regulators, modulate various physiological processes in numerous diseases including cancer. Upregulation or downregulation of miRNAs can affect the production of pro-oncogenic or anti-oncogenic proteins, influencing cellular processes that maintain tissue homeostasis and promote either apoptosis or differentiation, even in cancer cells. In order to understand the dysregulation of miRNAs, it is essential to examine miRNA biogenesis and any possible alterations at each step. The potential of a miRNA as a biomarker in prognosis, diagnosis, and detection is being assessed using technologies such as next-generation sequencing. Extensive research has explored miRNA expression profiles in cancer, leading to their utilization as diagnostic tools and the development of personalized and targeted cancer therapies. This review delves into the role of miRNAs in carcinogenesis in relation to the WNT signaling pathway along with their potential as druggable compounds.

## 1. Introduction

Carcinogenesis as a process is the progressive accumulation of alterations in the histology, biochemistry, epigenetics, and genetics of a particular organ. Multiple reports describe the heterogeneous nature of cancers, which aids in understanding resistance to therapy, progression of cancer, metastasis, and relapse. Numerous malignancies were investigated and cancer stem cells (CSCs) were one of the most peculiar cell types isolated. CSCs are believed to contribute to the pathogenesis of cancer due to their characteristic properties [1].

Several intriguing observations have been made from research on somatic stem cells and CSCs. An important characteristic of both these types of cells is self-renewal; however, in somatic cells, this mechanism is highly regulated. Mature cells arise from stem cells to produce normal healthy tissues whereas CSCs multiply to produce abnormal tissues. Here, the plasticity and heterogeneity of CSCs are observed, which are significant characteristics. Additionally, the molecular mechanism of stem cell mobilization to or from the niche is exploited by CSCs for invasion and metastasis. While addressing issues of metastasis and recurrence of cancer, characteristics of CSCs are of particular importance. These traits mainly include stemness, resistance to chemotherapy, and invasiveness amongst others [2]. 

An imperative and a highly conserved signaling pathway that contributes to the maintenance of pluripotency in somatic stem cells is the WNT signaling pathway. In cancer, an upregulation of WNT signaling has been observed, which promotes the WNT downstream protein β-catenin and its binding to lymphoid enhancer-binding factor 1 (LEF1) in the nucleus. This increase has been shown to correspond to the recurrence, metastasis, invasion, and progression of cancer, thus leading to low survival outcomes. Studies have also reported increased WNT signaling in CSCs as compared to bulk cancer cells [3]. 

CSCs can be better understood by the study of the regulation of molecular mechanisms involving microRNAs (miRNAs). miRNAs are post-transcriptional regulators of gene expression. They are typically 20–24 nucleotides long and cause gene silencing by binding to the 3′ untranslated region (UTR) of an mRNA. Several miRNAs are involved in the regulation of cellular activities to achieve a desirable physiological state. Consequently, the involvement of miRNAs in the dysregulation of these processes in diseases such as acquired immune deficiency syndrome (AIDS), diabetes, hypertension, and cancer has been reported [2]. Numerous tumor suppressor genes and oncogenes are targets of miRNAs and hence affect the persistence and progression of cancer. These miRNAs act through various signaling pathways responsible for chemoresistance, metastasis, epithelial to mesenchymal transition (EMT), angiogenesis, and stemness. Upregulation or downregulation in expression of any of these regulator miRNAs may cause a modulation in the pro-oncogenic or anti-oncogenic protein expression [4]. Reports have established the importance of miRNAs in the regulation of several cellular processes to maintain tissue homeostasis. The process of differentiation is often disrupted in cancer cells to promote the existence of phenotypes that allow for cancer progression and miRNAs have a crucial role to play in such a disruption [5]. miRNA dysregulation has been observed in another major concern, that is, therapeutic resistance. Expression profiling studies have established that CSCs rarely express tumor suppressor miRNAs but exhibit the enhanced expression of oncogenic miRNAs. These include miR-215, miR-181, miR-9, and LIN28B, which have been shown to influence chemoresistance and tumor initiation observed in CSCs. Since these miRNAs regulate specific genes to induce the chemoresistant phenotype, CSCs can be specifically targeted to enhance chemosensitivity and thereby prevent cancer relapse [6]. There have been increasing studies on the topic of miRNAs in carcinogenesis. These studies have explored the expression profiles of miRNAs in cancer, use of miRNAs as diagnostic tools, and miRNAs for personalized and targeted cancer therapies [5]. 

This review discusses the role of miRNAs in carcinogenesis and their contribution in facilitating stemness properties in CSCs. In addition to this, miRNAs, specifically in the context of the WNT signaling pathway in CSCs, will be discussed. Finally, the value of miRNAs as therapeutics will also be touched upon. 

## 2. Implication of miRNAs in Cancer

The presence of a malignancy can cause the dysregulation of miRNAs. This was first observed in a study on chronic lymphocytic leukemia where the absence of two miRNA genes, miR-15a and miR-16-1, was attributed to the deletion of chromosome 13q14. As a consequence, a variety of human cancers were studied to obtain a miRNA gene map. A significant number of miRNAs were found to be located in regions of the chromosome that were prone to deletions or amplifications [7]. As miRNAs are situated in the more unstable sites of the genome, they are prone to genetic modulations resulting in a higher occurrence of dysregulation. These modulations may include translocations, amplifications, deletions, and point mutations. Another factor contributing to miRNA dysregulation includes DNA hypomethylation and hypermethylation. Aberrations of this nature affect the levels of miRNAs in normal tissue and tumor tissue. Hence, the expression levels of miRNAs either increase or decrease in cancer. Among these, the miRNAs that target tumor suppressor gene expression are known as oncogenic miRNAs and those that target oncogene expression are known as tumor suppressor miRNAs [8]. 

There are numerous mechanisms by which the dysregulation of miRNAs occurs. This includes abnormalities in the genome, epigenetic alterations, regulation at the transcriptional level, and aberrations in the miRNA biogenesis pathways. 

### 2.1. Genome Abnormalities

Mutations at the location of the genes and copy number of miRNAs lead to an aberrant miRNA expression level in tumor cells as opposed to normal cells. It has been reported that the increased chromosomal instability in genes encoding miRNAs can be attributed to its innate repetitiveness [9]. The transcription of approximately half of the miRNAs occurs from the intergenic region and the introns account for around 40% of the miRNAs transcribed. The transcription of miRNAs from the introns is simultaneous with the host genes whereas the transcription of intergenic miRNAs is not well understood. The tumorigenic regions of the genome or the regions adjoining fragile sites contain several miRNAs [10]. 

A classic example of gene deletion affecting the expression levels of miRNAs is seen in B-cell chronic lymphocytic leukemia. In this condition, the miR-15a/16-1 cluster present in chromosomal region 13q14 is often deleted, thereby resulting in the reduced expression levels of miR-15 and miR16 [11,12]. Another instance of gene deletion leading to miRNA dysregulation has been indicated in epithelial ovarian cancer. It has been observed that 15% of all gene deletions contribute to the downregulation of miRNAs in epithelial ovarian cancer [9]. A group of researchers characterized and distinguished pineoblastoma tumors according to their clinico-pathological features. DNA methylation profiling and miRNA sequencing were the techniques used for this study. Out of the cases explored, two-thirds of the cases showed mutations in enzymes facilitating miRNA biogenesis. These enzymes include DROSHA, DGCR8, and DICER1 and the nature of the mutations was eminently focal deletions [13]. Let7D and miR-205 are highly conserved miRNAs that were often found to be mutated in the pan-cancer analysis. In addition to miRNAs, miRNA targets and miRNA sponges that target miRNAs to suppress their expression can also be mutated in malignancies. Deletions at the 3′UTR of targets, which is a binding site for miRNAs, may also arise due to mutations. Single-nucleotide polymorphisms (SNPs) present in the 3′UTR of targets can also compromise the binding of a miRNA to its target. Consequently, the complementarity of a miRNA and its target is compromised, leading to a lack of target inhibition. An instance of this is a mutation seen in E2F1, which is an oncogene. There is a loss of target inhibition as miR-136-5p is unable to bind to the mutated 3′UTR of E2F1. As a result, a malignant phenotype is observed due to an upregulation in the expression of E2F1 [14]. 

### 2.2. Epigenetic Alterations

In cancer, epigenetic alterations such as destabilizing patterns of histone modifications, atypical hypermethylation at sites of tumor suppressor genes, and global hypomethylation of genomic DNA are commonly observed. Epigenetic alteration might also occur at sites in the genome coding for miRNAs comparable to protein-coding sequence alterations [11]. 

A study on epithelial ovarian cancer described that the dysregulation of transcription throughout the genome can be attributed to a reduction in the expression levels of miRNAs. Epigenetic silencing and loss in genome copy number is responsible for a reduction in miRNA levels by approximately 15% to 36%. One of the miRNAs that p53 regulates is miR-34b, a tumor suppressor miRNA, which was found to be downregulated due to epigenetic silencing in epithelial ovarian cancer. Situated at the Dlk1-Gtl2 domain of chromosome 14 is a cluster of tumor suppressor miRNAs. Epigenetic silencing at this domain resulted in the downregulation of eight prominent tumor suppressor miRNAs in the case of late-stage epithelial ovarian cancer. Consequently, there was an increase in the levels of their target mRNAs, which are involved in carcinogenesis, as demonstrated by gene ontology analysis [15]. Another instance of epigenetic silencing was demonstrated by a study executed on breast cancer cells. The expression of a majority of tumor suppressors is inhibited by hypermethylation at the promoter region. This study illustrated that the miR-193a-3p promoter was hypermethylated in the case of breast cancer and the amount of methylation coincided with the stage of cancer as well as grade. Normally, miR-193a-3p targets various aspects of carcinogenesis such as the migration, invasion, and proliferation of cells in breast cancer [16]. Studies on lung cancer have implicated reduced expression levels of the miR-29 tumor suppressor [17]. Due to this, an abnormal pattern of DNA methylation is observed as this downregulation causes an increase in DNA methyltransferase expression. On establishing a stable miR-29 expression, the normal DNA methylation patterns are reinstated. Thus, this allows for the expression of the tumor suppressor genes that were previously methylated, resulting in the inhibition of cancer progression [18]. 

### 2.3. miRNA Regulation at the Transcriptional Level

**Figure 1:**
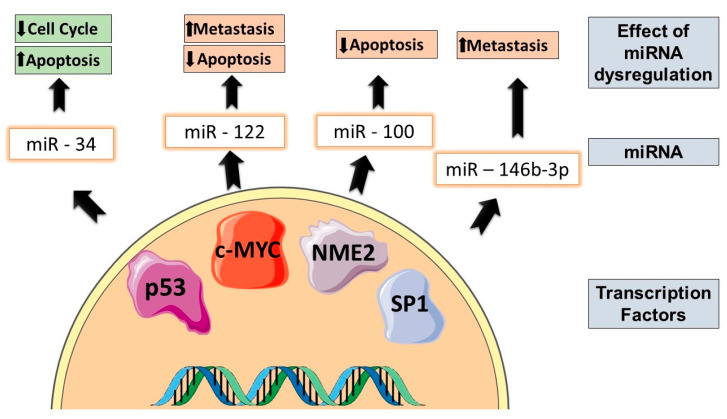
Transcription factors influencing the dysregulation of specific miRNAs resulting in a change in tumor cell properties. The up and down arrows are representative of the promotion and inhibition of the tumor cell property, respectively.

Various transcription factors regulate the expression of miRNAs and the dysregulation in the miRNA expression may be due to an alteration in these transcription factors as illustrated in Figure 1. In order to effectively regulate the expression of genes and signaling pathways, miRNAs and transcription factors co-regulate one another. Several studies have observed that a significant population of miRNA targets are transcription factors [19]. The transcription factors that typically regulate miRNAs in malignancies are p53 and c-Myc, as reported by various studies. In cancer, TP53, the gene encoding p53, is frequently mutated. miRNAs that are regulated by p53 form a network to modulate pathways of apoptosis and cell cycle progression. The expression of a wide variety of miRNAs such as miR-107, miR-1246, and miR-605 are regulated by p53. miR-34 is a tumor-suppressive miRNA that induces apoptosis, senescence in cells, and cell cycle arrest in cancer. Studies further demonstrated that the miR34a expression is regulated by p53 via the binding of p53 to the miR-34a promoter region. Additionally, SIRT1 is a target of miR-34a, which negatively regulates the expression of p53 through deacetylation [11]. An important feedback loop in carcinogenesis is the regulation of miR-122 by c-Myc through promoter binding. Additionally, miR-122 indirectly suppresses E2F1 and TFDP2, which leads to c-Myc transcription inhibition. The significance of c-Myc is demonstrated by the tumor suppressor miRNAs that it regulates, such as let-7, miR-30, miR-29, miR-26, and miR-15a [20]. 

A report on gastric cancer cells showed that the transcription factor NME2 could regulate miR-100 transcription along with LIMS1, STARD5, and RIPK1, all of which function to counteract apoptosis. It has also previously been shown that targeting the inhibition of miR-100 can lead to the inactivation of p53 degradation via ubiquitination, thereby inducing gastric cancer cells to undergo apoptosis [21]. miR-100 directly targets a E3-ubiquitin ligase known as RNF144B. Under cell-damaging events, RNF144B promotes apoptosis via p53 degradation [22]. miR-146b-3p was investigated in the context of colorectal cancer. In this study, SP1 was ascertained as a critical transcription factor influencing miR-146b-3p expression by binding to its promoter. An overexpression of SP1 greatly enhanced the expression of pro-oncogenic miR-146b-3p. This overexpression is associated with the invasion, migration, and proliferation of colorectal cancer cells. Additionally, this study also demonstrated that downregulating the expression of miR-146b-3p via SP1 can overrule these effects [23]. 

### 2.4. Aberrant Biogenesis of miRNA

**Figure 2:**
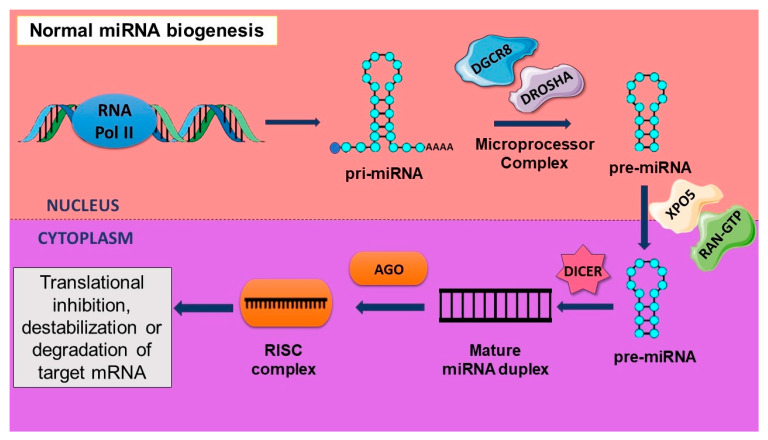
Normal process of miRNA biogenesis leading to translational inhibition, destabilization, or degradation of target mRNA.

The activity of different enzymes at the transcriptional and post-transcriptional level contributes to the biogenesis of miRNAs affecting their expression levels. A stem–loop structure of a pri-miRNA with a 5′ end cap and a poly(A) tail at the 3′end results after the transcription of miRNA genes by RNA polymerase II; however, RNA polymerase III transcribes a small population of miRNAs. Ribonuclease reactions are at the core of the canonical biogenesis pathway, which consists of two steps occurring in the nucleus and the cytoplasm. The microprocessor complex, consisting of an RNase III enzyme DROSHA and a double-stranded RNA binding protein DGCR8, processes the pri-miRNA into a precursor miRNA with a hairpin structure. DGCR8 recognizes the junction between the two strands of the pri-miRNA to allow for DROSHA to liberate the pre-miRNA. The 70-nucleotide-long pre-miRNA is transported to the cytoplasm from the nucleus by Exportin-5 (XPO5) along with Ran-GTP. Another RNase III enzyme known as Dicer associates with a few other proteins to further process the pre-miRNA, in the cytoplasm, into a mature miRNA duplex, which is 20–25 nucleotides in length. Out of the two strands of the duplex, one of them is loaded onto the Argonaute (AGO) protein to form the RISC- or RNA-induced silencing complex. Normally, the second strand of the duplex is degraded. As a part of the RISC complex, miRNA acts as a guide to target mRNAs for translational inhibition, destabilization, or degradation by the AGO protein [24]. The process of miRNA biogenesis has been illustrated in Figure 2.

Dysregulation of mature miRNA levels may also be due to improper pre-miRNA nuclear export in cancer cells. It was also shown that there is a retention of pre-miRNAs in the nucleus of tumorigenic cells as compared to normal cells. The expression profiling of 225 pre-miRNAs in normal tissues as well as cancer cell lines established that this retention of pre-miRNA does not allow for mature miRNA processing, and this may be due to an aberration in the process of export from the nucleus via XPO5. A reduction in the levels of XPO5 due to genetic or epigenetic modulation and XPO5 post-translational modifications may be the reason behind this defect [25]. Studies have also discussed the downregulation of DROSHA and DICER in cancer. There is an improved risk of cancer if a germline mutation is detected in any of the genes involved in miRNA biogenesis. In pleuropulmonary blastoma, it has been frequently observed that DICER1 undergoes a heterozygous germline mutation. These mutations result in a truncated protein close to the two carboxy terminal functional domains of RNase III of DICER1. Similarly, in ovarian cancer, the functional domains of RNase undergo a somatic mutation. Although in this case the protein function was not totally lost, the activity of RNase III was alleviated. An increased rate in the proliferation of cancer cells of the lung adenocarcinoma was observed due to DROSHA knockdown [14].

**Figure 3:**
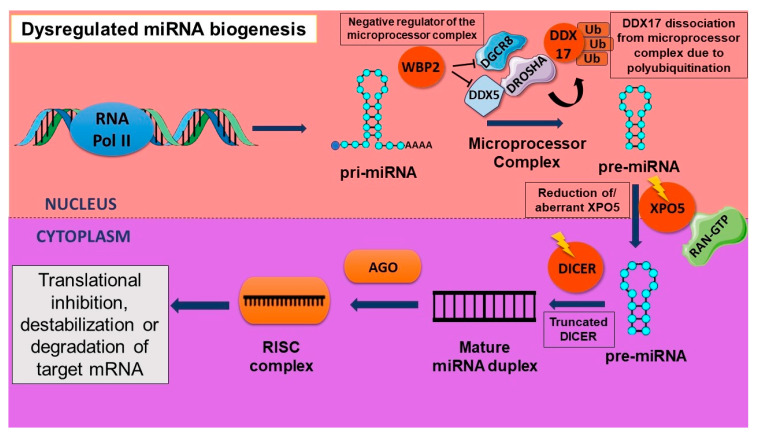
Dysregulation of miRNA biogenesis resulting due to aberrant protein components of the miRNA biogenesis machinery. Aberrant proteins such as dysregulated WBP2, polyubiquitinated DDX17, mutated XPO5, and truncated DICER protein cause a reduction in processing of miRNA at various points in the biogenesis affecting their expression levels.

The microprocessor complex is negatively regulated by WBP2, a transcriptional coactivator. WBP2 promotes carcinogenesis in breast cancer by regulating important cellular pathways. However, this protein is regulated by miRNA processing machinery as it is involved in the production of miRNAs that target WBP2. In breast cancer, physical binding of WBP2 with the components of the microprocessor complex such as DDX5 and DGCR8 led to a reduction in pri-miRNA processing. This resulted in an increase in the oncogenic activity via the increased proliferation of breast cancer cells [26]. Tumor suppressor miR-145 targets SOX9 and TWIST to prevent ovarian CSC formation. A PI3K/AKT pathway effector, p70 S6 kinase (p70S6K), targets the miRNA biogenesis pathway components Dicer and tristetraprolin (TTP), leading to a decrease in the maturation of miR-145. This study also discussed that a reduction in the expression levels of TTP correlates with an increase in p70S6K. This dysregulation may be one of the essential causes of poor prognosis in ovarian cancer [27]. A transcriptional coactivator and microprocessor complex cofactor—DDX17—has been shown to modulate CSC properties. When DDX17 is polyubiquitinated by HectH9, an E3 ligase, it dissociates from the microprocessor complex, which is facilitated by nuclear YAP. This results in a reduction in tumor suppressor miRNA biogenesis and promotes the stemness properties of CSCs. Previous studies have discovered a site at the 3′end of the pri-miRNA containing a specific sequence motif that can be identified by DDX17. This study demonstrated that DDX17 polyubiquitination interferes with its binding to tumor suppressor miRNAs, which contain the specific sequence motif for it, thus resulting in the downregulation of tumor suppressor miRNA biogenesis. In addition to this, a YAP-DDX17-p300 complex results as polyubiquitinated DDX17 improves the binding of p300 to it. Under a hypoxic condition, there is an increase in the acetylation of H3K56 in genes related to stemness, which coincides with the formation of the YAP-DDX17-p300 complex. Hence, a transcriptional activation of stemness genes has been observed in these cells [28]. Figure 3 illustrates the involvement of aberrant protein components in the dysregulated miRNA biogenesis.

## 3. How Are Stemness Properties in CSCs Related to miRNAs?

The quiescent nature of a cancer, its initiation, and progression have been attributed to CSCs. The main concern of low survival rates in cancer is persistent despite the emergence of new treatment regimes. Reports indicate that most often, treatments augment the population of CSCs, and this is due to the properties of initiation of cancer, self-renewal, and drug resistance in these cells. When the status of miRNAs was studied in these cells, it was found that some of them were dysregulated. This gave incentive to the study of miRNAs in various malignancies [29].

A group of researchers studied liver cancer stem cells in the context of hepatocellular carcinoma and demonstrated the effect of the miRNA-302a/d in suppressing the CSC properties such as CSC self-renewal. Experiments such as tumor formation in vitro and in vivo and cell proliferation were carried out to determine the stem-like potential of liver CSCs. A low expression level of miR-302a/d in liver cancer stem cells and an inhibition of the CSC properties on increasing its levels indicated its function as a tumor suppressor miRNA [30]. Another group investigated the properties of tumor initiation, self-renewal, and resistance to chemotherapy of colorectal cancer stem cells and its relation to miR-451. The expression of miR-451 was found to be low in these CSCs compared to tumor bulk cells. Cyclooxygenase-2 (COX-2) contributes to the growth of CSC spheres. This study showed that COX-2 is an indirect target of miR-451 and the upregulation of this miRNA caused a decrease in the CSC sphere formation [31].

Multiple reports discuss the influence of miRNAs in the chemoresistance of CSCs. A study on pancreatic CSCs that were resistant to gemcitabine demonstrated the involvement of miR-210 in drug resistance. On isolation of chemotherapy-resistant and sensitive populations of cells, the expression of proteins related to chemoresistance such as BCRP, YB-1, and MDR were found in the resistant population. Further, when exosomes isolated from the culture medium of the chemoresistant population were cultured with the chemosensitive population, the chemoresistance of these cells to gemcitabine increased significantly. On examination of these exosomes, it was found that miR-210 was highly expressed. Overexpression of this miRNA has shown progression of cancer along with chemoresistance. It was concluded that a chemoresistant population of cells can deliver miR-210 through exosomes to promote chemoresistance in chemosensitive populations [32]. A tumor suppressor miR-485 was found to facilitate sensitivity to chemotherapy in non-small-cell lung cancer CSCs. CD44, a prominent CSC marker, is a target for miR-485. Subsequently, an increase in the expression levels of miR-485 by the epigallocatechin-3-gallate compound resulted in a decrease in CD44. Hence, a decrease in CSC properties such as chemoresistance was observed [33].

A breast cancer study used MCF-7 cells to generate CSC spheroids and analyzed the difference in the expression levels of miRNAs in parental cells and CSCs using next-generation sequencing. These CSCs demonstrated properties of chemoresistance, self-renewal, migration, increased proliferation, and expressed CSC markers. Novel miRNAs that contribute to breast CSC chemoresistance and self-renewal have been reported in this study, which included miR-365a, miR-1296, miR-4448, miR-4508, miR-381, miR-4532, and miR-4492. Gene ontology analysis revealed that these miRNAs were involved in the regulation of different pathways involving metastasis, tumor initiation, chemoresistance, and self-renewal in breast cancer [34]. In nasopharyngeal carcinoma, low expression levels of miR-183 were observed. miR-183 overexpression resulted in a reduction in stemness markers, cell proliferation, and in vivo tumor formation. CSCs were specifically targeted as the sphere formation was inhibited in CSCs expressing miR-183. The decrease in the stemness properties was due to the repression of the SOX2 and OCT4 expression levels in miR-183 expressing CSCs. Notch signaling was also observed to be downregulated in these CSCs expressing miR-183, further confirming its essentiality in regulating the stemness properties in nasopharyngeal carcinoma [35].

## 4. Eminent miRNAs in Specific Malignancies

Dysregulated miRNA expression levels and signaling pathways have been shown to contribute to the progression of cancer. The regulation of miRNAs is crucial since their roles can be either pro-oncogenic or tumor-suppressive. The targets of miRNAs govern their oncogenic potential, and their targets are involved in numerous cellular processes like apoptosis, angiogenesis, migration, differentiation, cell cycle, and proliferation. This is summarized in Table 1.

## 5. Prominence of miRNAs in WNT Signaling Pathway Dysregulated in CSCs

**Figure 4:**
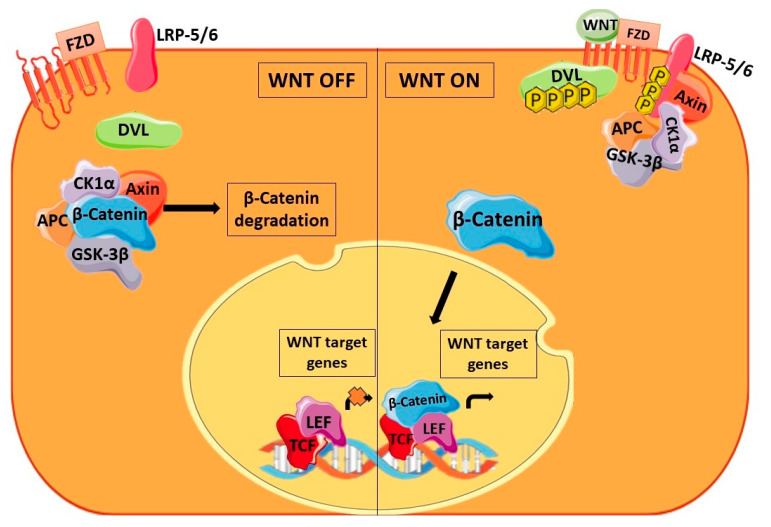
General overview of the WNT signaling pathway. The figure represents the WNT signaling pathway in the absence/presence of the WNT ligand. The absence of the WNT ligand causes the degradation of β-catenin via the destruction complex whereas in the presence of the WNT ligand, β-catenin activates the transcription of the WNT target genes.

An abnormal activation of the WNT signaling pathway is observed in cancer stem cells of different malignancies. The canonical WNT pathway shown in Figure 4 is activated on binding of WNT ligands to Frizzled (FZD) receptors. On binding of the ligand, the co-receptor low-density lipoprotein receptor-related protein-5/6 (LRP-5/6) is phosphorylated and there is subsequent phosphorylation of Dishevelled (DVL), which is recruited to the plasma membrane. In the cytoplasm, the destruction complex consisting of glycogen synthase kinase 3β (GSK-3β), casein kinase 1α (CK1α), adenomatous polyposis coli (APC), and Axin sequesters β-catenin and causes its degradation. Once DVL is phosphorylated, β-catenin is released from the destruction complex. The unbound β-catenin can now translocate to the nucleus and enable the transcription of target genes along with T-cell factor/lymphoid enhancer (TCF/LEF) transcription factors. Multiple CSC properties are regulated by these target genes, highlighting the importance of this pathway in CSCs. Also, the growth of CSCs has been correlated with β-catenin overexpression [50]. Several reports are focusing on the regulation of the WNT pathway by miRNAs. miRNAs can induce or suppress the WNT pathway at various points by targeting its receptor/ligand as well as the associated proteins, the destruction complex, β-catenin, and the transcription factors.

### 5.1. At the Extracellular Level of WNT Signaling

A study described the role of miR-600 in determining the fate of breast CSCs. The tumorigenicity, in vivo, was assessed by overexpressing or silencing miR-600, which resulted in the expansion of CSCs and a decrease in tumorigenicity, respectively. The enzyme involved in producing lipid-modified, active WNT proteins—stearoyl desaturase 1 (SCD1)—was identified as the target of miR-600. It was, therefore, inferred that activation of WNT signaling resulted in miR-600 silencing, leading to CSC expansion, whereas the inhibition of WNT signaling resulted when miR-600 was overexpressed, leading to decreased tumorigenicity in vivo. The researchers correlated these results with 120 breast cancer cases. Regulation of the WNT pathway by miR-600 targeting SCD1 is imperative in determining breast CSC fate. This study concludes that CSC expansion is highly dependent on lipid-modified WNT production [39]. Another miRNA studied in the context of the WNT pathway in breast cancer is miR-148a. WNT-1 is an immediate target of miR-148a, which is one of the WNT pathway ligands. The results of the overexpression of miR-148a on breast CSCs showed that not only WNT-1 mRNA and protein expression but significant components of the pathway such as β-catenin, TCF-4, and matrix metalloproteinase-7 (MMP-7) were also downregulated. The targeting of WNT-1 by miR-148a consequently resulted in the reduction in WNT signaling and an inhibition in the migration and invasion of breast CSCs [40]. Microarray analysis of gastric cancer-associated myofibroblasts (CAMs) showed an increase in the expression levels of miR-181d. On further investigation of the microarray dataset, the non-canonical WNT pathway ligand WNT5a showed a sustained increase in expression in gastric CAMs. miR-181d knockdown resulted in WNT5a downregulation as well as the reduced proliferation and migration of gastric CAMs. Additionally, such miRNA-level dysregulation contributes to the CAM effect on CSC migration. This study, therefore, highlighted the role of miR-181d and WNT5a in the proliferation and migration of gastric CAMs [43]. A study on colorectal cancer showed the downregulation of miR-375, which was associated with possible metastatic traits in a tumor. The knockdown of miR-375 in these cells caused enhanced migration and invasion whereas the upregulation of miR-375 had an inverse effect. On investigation of the molecular mechanism, it was found that an immediate target of miR-375 is FZD8. There was an inverse correlation of expression levels observed between miR-375 and FZD8. This study demonstrated downregulation in the expression of FZD8 on miR-375 overexpression. Consequently, there was a WNT pathway inhibition and reduction in colorectal cancer cell migration and invasion [44]. A study on ovarian CSCs demonstrated the hyperactivation of STAT3 and this was associated with the WNT pathway. On suppression of STAT3 in ovarian CSCs, dickkopf-1 (DKK1), which is a WNT pathway inhibitor, showed a significant upregulation in expression. This study also established that DKK1 silencing that is facilitated by STAT3 is via miR-92a. miR-92a binding to a specific sequence in the 3′ UTR of DKK1 mRNA thereby downregulates its expression. Certain regions in the promoter region of miR-92a are directly bound by STAT3, enabling the transcription of this miRNA. Thus, miR-92a plays an important role in regulating the WNT pathway by inactivating the WNT antagonist DKK1 in ovarian CSCs [36]. miR-217 was shown to be upregulated in hepatocellular carcinoma CSCs. This CSC phenotype manifested due to the targeting of DKK1 by miR-217 led to constitutive WNT signaling activation. To show that miR-217 directly targets DKK-1, the researchers demonstrated that in miR-217-overexpressing cells, WNT antagonism and reduction in stemness was observed on DKK-1 overexpression. A converse effect was observed when DKK-1 was silenced in miR-217-downregulated cells [45].

### 5.2. At the β-Catenin Level

A study on ovarian cancer demonstrated that miR-506-3p directly targets EZH2 in ovarian CSCs, which has a repressive effect on chemoresistance. EZH2 has been implicated to be significantly involved in multidrug resistance. Additionally, the β-catenin pathway is activated by EZH2. Overexpression of miR-506-3p showed a downregulation in β-catenin expression levels. This regulation leads to a decrease in chemoresistance in ovarian CSCs [38]. Tumor suppressor miR-133b in lung cancer was associated with the WNT pathway and SOX9. Normally, miR-133b is downregulated in lung cancer whereas on overexpression, it impedes cancer cell proliferation and invasion and increases cellular apoptosis. Upregulation of miR-133b also resulted in a decreased expression of SOX9 and β-catenin. On upregulation of SOX9, there was a disruption of cellular apoptosis caused due to miR-133b upregulation. An increase in the activity of the WNT pathway is seen due to the positive regulator of LRP6 and TCF4, SOX9. This leads to a decrease in apoptosis and increase in cell proliferation, emphasizing the importance of the SOX9/WNT pathway in lung cancer [49]. Oncogenic miR-766 is implicated in hepatocellular carcinoma to cause tumor progression and metastasis. miR-766 directly targets NR3C2 to induce the proliferation and metastasis of cells. The transcription factor NR3C2 regulates the activation of target genes by binding to mineralocorticoid response elements. Targeting of NR3C2 by miR-766 led to modulation in the WNT pathway. The study demonstrated that a reduction in NR3C2 levels due to miR-766 causes an upregulation of β-catenin [46].

### 5.3. At the Destruction Complex Level

A study on liver CSCs demonstrated that miR-1246 activates the WNT pathway, thereby enhancing the stemness properties including tumorigenicity, chemoresistance, self-renewal, and metastasis. miR-1246 targets components of the destruction complex—AXIN2 and GSK3β—which leads to the activation of the WNT pathway. This study also established the existence of the OCT4/miR-1246 signaling axis wherein OCT4 is the upstream regulator of miR-1246. A disruption in the assembly of the destruction complex is observed when there is an upregulation of miR-1246, enhancing the WNT pathway. β-catenin mutation has been frequently observed in hepatocellular carcinoma. In the absence of this type of mutation in certain samples, the role of miR-1246 in the activation of the WNT pathway has been established [47]. In breast CSCs, the WNT pathway has been shown to be activated when miR-142 targets APC, which in turn causes miR-150 expression. Tumor progression was significantly attenuated on endogenous miR-142 knockdown. A reduction in the APC expression levels and increased WNT signaling due to miR-142 was shown in malignant mammary epithelium. miR-150 contributes to cellular proliferation and is regulated downstream of the WNT pathway. This was confirmed as the miR-150 promoter region harbors a binding site for TCF/β-catenin [41].

### 5.4. At Transcriptional Level

A study on pancreatic CSCs showed the implication of miR-137 on the stemness properties of CSCs. In this study, the activation of the WNT pathway was observed as miR-137 targets the KLF12 transcription factor. In miR-137-overexpressing and consequently KLF12-downregulated cells, there was a considerable reduction in WNT signaling whereas an increase was observed in the case of miR-137 inhibition. On investigation of signaling pathways downstream of KLF12, it was found that there was a strong association of this transcription factor with the WNT pathway. On further analysis, it was found that the expression of KLF12 correlates with DVL2. It was also shown that KLF12 transcriptionally modulates DVL2 expression, thereby leading to the constitutive activation of the WNT pathway [48]. The status of miR-195 was investigated in colorectal CSCs and was found to be downregulated. On miR-195 overexpression, the G2/M phase of the cell cycle was arrested, and the viability of the cells reduced significantly. The direct target of miR-195 is FGF2, which rescues reduced colorectal CSC viability and CDK2, CyclinD1, CyclinB1 downregulation caused by miR-195 overexpression. Cell proliferation and activation of target genes such as CyclinD1 is regulated by β-catenin. Overexpression of miR-195 also showed the upregulation of GSK-3β and the downregulation of TCF4, LEF1, and β-catenin, indicating its role in the regulation of cell proliferation through the WNT pathway [51].

**Figure 5:**
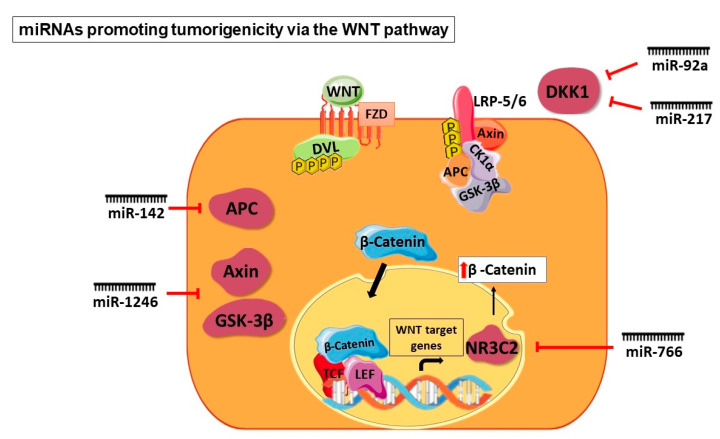
miRNAs targeting different components of the WNT signaling pathway to promote tumorigenicity. The pro-oncogenic miRNAs like miR-92a, miR-217, miR-766, miR-1246, and miR-142 act on various levels of the WNT pathway to promote tumorigenicity. More commonly, these miRNAs negatively regulate the components of the destruction complex and WNT antagonists and promote WNT ligand production, thereby enabling the WNT signaling pathway.

**Figure 6:**
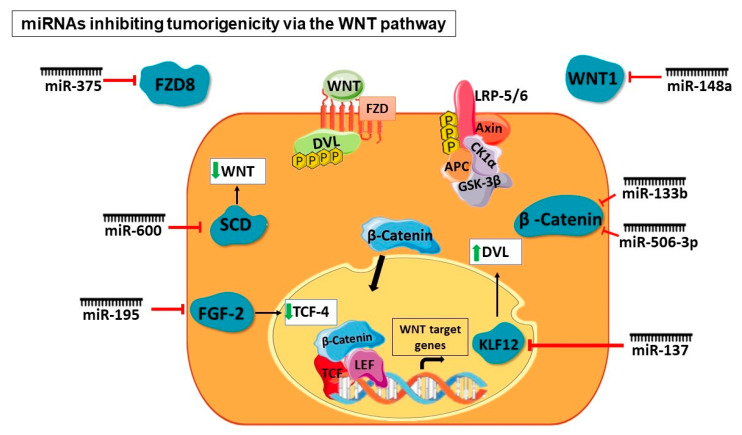
miRNAs targeting different components of the WNT signaling pathway to inhibit tumorigenicity. The tumor suppressor miRNAs like miR-375, miR-148a, miR-133b, miR-506-3p, miR-137, miR-195, and miR-600 act on various levels of the WNT pathway to inhibit tumorigenicity. These miRNAs negatively regulate the WNT signaling pathway components such as the receptors, ligands, β-catenin, and transcription factors.

The miRNAs promoting or inhibiting tumorigenicity at different levels of the WNT signaling pathway have been illustrated in Figure 5 and Figure 6.

## 6. miRNA as Druggable Compounds

A substantial part of cancer regulation has been attributed to miRNAs, making them essential therapeutic alternatives. In vitro and in vivo experiments have provided the grounds for the entry of miRNAs in the clinical scenario. miRNAs have been used as therapeutic targets or therapeutic agents, which require either their inhibition in target cells or delivery to target cells, respectively. Replacement therapy is used on the loss or repression of miRNA expression where a double-stranded RNA mimic of the miRNA is administered to cause the appropriate cancer phenotype suppression. Antisense binding sites of the target miRNA are present in the miRNA sponge, thereby acting as suppressors of the target miRNA [52]. Another miRNA inhibition technique is the utilization of antisense oligonucleotides where they degrade the target miRNA by inducing RNase activity [53].

Two categories of delivery, viral or non-viral, are available, which are selected based on the desired outcome. Replacement and overexpression of miRNAs using viruses as the delivery system have majorly been reported in in vitro studies. As far as miRNA sponges are concerned, in vivo studies require delivery through viral vectors that are systemically injected. Non-viral delivery systems consist of nano-carriers that are polymer- or lipid-based. The basis of this technology is the use of positively charged, biodegradable carriers for the negatively charged miRNA and the resulting electrostatic complex between the two. Antisense oligonucleotides or synthetic miRNAs are introduced in these carriers in the case of replacement therapy [52].

Our lab has studied the effect of RNAi technology on AD201, a WNT antagonist, in the context of the Alzheimer’s disease model. As opposed to cancer, neurodegeneration usually observes the downregulation of WNT signaling. In this model, it was observed that using RNAi technology on AD201 rescues the neurodegeneration phenotype via the activation of WNT signaling. This involved a decrease in oxidative stress, improvement in mitochondrial function, and rescue of neuronal function. This emphasizes the importance of such a technology in therapy [54]. It has been reported in ovarian cancer that let-7d-5p targets CSC characteristics to subdue cancer progression. Let-7d-5p targeted HMGA1 in ovarian CSCs to enhance p53 signaling. This resulted in decreased proliferation and chemoresistance and increased cellular apoptosis. This suggests that let-7d-5p could be further employed as a therapeutic agent [55]. An inverse correlation between the expression levels of miR-613 and FZD7 was observed in clinical prostate cancer samples. Downregulation of WNT signaling on the overexpression of miR-613, which acts via targeting FZD-7, was observed. Consequently, there was decreased proliferation and metastasis of these cells. The therapeutic potential of miR-613 has been highlighted in this study [56].

The stability and sustainability of miRNAs in circulation is one of the biggest obstacles for miRNAs to be used as treatments. RNase A present in the serum will degrade miRNAs with the ribose moiety containing an unmodified 2′OH in a few seconds. In systemic circulation, the short half-life of these miRNAs is dictated by renal excretion. On modifying 2′ribose and the phosphodiester backbone, the miRNAs last longer in systemic circulation and are more stable. Additional modifications in chemically engineered miRNAs include 2′-Fluoro, 2′-locked nucleic acid, 2′-O-Methyl, 2′-O-(2-Methoxyethyl) ribose backbone, and phosphodiester linkages. The stability of the oligonucleotide is enhanced through these modifications along with improved affinity for the target, thereby maximizing the overall performance of the miRNA [57]. miRNA-based therapeutics have been primarily administered via the skin or intravenous route. Another route that has been tested clinically for RNA-based therapies is inhalation for respiratory diseases. Investigation on more extensive protocols is underway for miRNA-based therapies. A biodegradable 3D matrix containing miRNA drugs is a potential protocol that would involve implantation at the tumor site via surgery [58].

Formulated using liposomes, the miR-34 mimic, MRX34, was used as an investigational drug in clinical trials for the treatment of hepatocellular carcinoma. The preclinical studies, conducted using two different models of orthotopic liver cancer, suggested that this therapy enabled significant miR-34a delivery to the site of the tumor and curbed tumor growth. Additionally, MRX34 was tested for any upregulation in prominent immune cytokines in an immunocompetent liver tumor model. In the trial conducted by Mirna Therapeutics Inc., patients with recurrent solid tumors were enrolled (*n* = 47). However, due to severe immunological responses observed in five patients, the trial was terminated. A number of reasons have been cited for the immunological response. It could be due to the use of double-stranded oligonucleotides or the liposomal carrier. Additionally, the lack of a targeting molecule along with the mimic for a more specific delivery could also be a reason as the mode of administration was intravenous [59,60]. There is an attempt to identify inherent transcripts that can act as miRNA sponges in therapies. hsa_circ_0120472, a circular RNA, has been recently tested as a miRNA sponge, which has two binding sites for miRNAs. In breast cancer cells, hsa_circ_0120472 has been shown to effectively inhibit miR-550a [58]. In hepatitis C virus infection, miR-122 is involved in enhancing viral replication by binding to the 5′untranslated region of the viral genome. Miravirsen^®^—a 15-nucleotide-long locked nucleic acid (LNA)—which is an antisense oligonucleotide, was developed by Santaris Pharma A/S, San Diego, CA, USA to target mature miR-122. This clinical trial was successful with undetectable levels of the viral load in 5 patients out of 36 patients, which showed reduced levels of HCV with an increasing dose in phase IIa trials. The predominant adverse effects of this trial were fatigue and a mild headache, which deemed the drug safe overall. This drug is now being tested on a larger population along with a longer follow-up for the adequate assessment of safety and efficacy [60].

## 7. Conclusions

The process of dysregulation in a particular malignancy may manifest through different paths of genome abnormalities, epigenetic alterations, transcriptional regulation of miRNA, and the aberrant biogenesis of miRNA. Several reports have also discussed the importance of miRNA regulation in normal cells and how their dysregulation encourages the occurrence of CSC properties, thereby facilitating cancer progression. miRNAs target each component of the WNT pathway, which is the key driver of cancer stemness, to either promote or subdue the pathway. Our lab is currently working to understand the effect of the tumor suppressor miR-203a on ovarian CSCs. Our data suggest that miR-203a is able to target CSC properties such as chemosensitivity, EMT, and apoptosis, which is in line with our WNT antagonism studies on ovarian CSCs. According to reports, miRNAs may be the way ahead for tackling cancer’s most serious recurrence challenge today. The preliminary research findings unquestionably point us in the direction of clinics. High specificity to target mRNA, low off-target effects, and an ability to simultaneously regulate the targets of numerous pathways make miRNAs a highly competent therapeutic tool. More insight into the potential toxicity, delivery strategies, and immune response of miRNA-based therapies is required. Optimum modifications of these therapeutic miRNAs hold tremendous promise for future therapeutics.

## Figures and Tables

**Table 1:** Pathways targeted by miRNAs in specific malignancies.

Cancer Type	miRNA	Type of miRNA	Pathway Involved	Reference
B-cell chronic lymphocytic leukemia	miR-15, miR16	Tumor suppressor	Apoptosis	[11,12]
Epithelial ovarian cancer	miR-34b	Tumor suppressor	Apoptosis, senescence in cells, and cell cycle arrest	[11,15]
	miR-92a	Oncogenic	Tumor initiation and metastasis	[36,37]
	miR-506-3p	Tumor suppressor	Chemoresistance	[38]
Breast cancer	miR-193a-3p	Tumor suppressor	Migration, invasion, and proliferation of cells	[16]
	miR-600	Oncogenic	Proliferation of cells	[39]
	miR-148a	Tumor suppressor	Migration and invasion	[40]
	miR-142	Oncogenic	Metastasis	[41]
	miR-150	Oncogenic	Cellular proliferation	[41]
	miR-195	Oncogenic	Chemoresistance and metastasis	[42]
Acute myeloid leukemia	miR-29	Tumor suppressor	Inhibition of cancer progression	[17,18]
Gastric cancer	miR-100	Oncogenic	Apoptosis	[21]
	miR-181d	Oncogenic	Proliferation and migration	[43]
Colorectal cancer	miR-146b-3p	Oncogenic	Invasion, migration, and proliferation	[22]
	miR-451	Tumor suppressor	Tumor initiation, self-renewal, and resistance to chemotherapy	[31]
	miR-375	Tumor suppressor	Migration and invasion	[44]
Liver cancer	miRNA-302a/d	Tumor suppressor	Cell proliferation and tumor sphere formation	[30]
	miR-217	Oncogenic	Cell proliferation, chemoresistance, and metastasis	[45]
	miR-766	Oncogenic	Metastasis and proliferation of cells	[46]
	miR-1246	Oncogenic	Chemoresistance, self-renewal, and metastasis	[47]
Pancreatic cancer	miR-210	Oncogenic	Chemoresistance	[32]
	miR-137	Tumor suppressor	Cell proliferation and invasion	[48]
Non-small-cell lung cancer	miR-485	Tumor suppressor	Chemoresistance	[33]
	miR-133b	Tumor suppressor	Cell proliferation, invasion, and apoptosis	[49]
Nasopharyngeal carcinoma	miR-183	Tumor suppressor	Cell proliferation, self-renewal and chemoresistance	[35]
